# Machine learning-based prediction of hospital prolonged length of stay admission at emergency department: a Gradient Boosting algorithm analysis

**DOI:** 10.3389/frai.2023.1179226

**Published:** 2023-07-28

**Authors:** Addisu Jember Zeleke, Pierpaolo Palumbo, Paolo Tubertini, Rossella Miglio, Lorenzo Chiari

**Affiliations:** ^1^Department of Electrical, Electronic, and Information Engineering Guglielmo Marconi, University of Bologna, Bologna, Italy; ^2^Enterprise Information Systems for Integrated Care and Research Data Management, Istituto di Ricovero e Cura a Carattere Scientifico (IRCCS) Azienda Ospedaliero—Universitaria di Bologna, Bologna, Italy; ^3^Department of Statistical Sciences, University of Bologna, Bologna, Italy; ^4^Health Sciences and Technologies Interdepartmental Center for Industrial Research (CIRI SDV), University of Bologna, Bologna, Italy

**Keywords:** emergency department, prolonged length of stay, machine learning, prediction, classification, regression

## Abstract

**Objective:**

This study aims to develop and compare different models to predict the Length of Stay (LoS) and the Prolonged Length of Stay (PLoS) of inpatients admitted through the emergency department (ED) in general patient settings. This aim is not only to promote any specific model but rather to suggest a decision-supporting tool (i.e., a prediction framework).

**Methods:**

We analyzed a dataset of patients admitted through the ED to the “Sant”Orsola Malpighi University Hospital of Bologna, Italy, between January 1 and October 26, 2022. PLoS was defined as any hospitalization with LoS longer than 6 days. We deployed six classification algorithms for predicting PLoS: Random Forest (RF), Support Vector Machines (SVM), Gradient Boosting (GB), AdaBoost, K-Nearest Neighbors (KNN), and logistic regression (LoR). We evaluated the performance of these models with the Brier score, the area under the ROC curve (AUC), accuracy, sensitivity (recall), specificity, precision, and F1-score. We further developed eight regression models for LoS prediction: Linear Regression (LR), including the penalized linear models Least Absolute Shrinkage and Selection Operator (LASSO), Ridge and Elastic-net regression, Support vector regression, RF regression, KNN, and eXtreme Gradient Boosting (XGBoost) regression. The model performances were measured by their mean square error, mean absolute error, and mean relative error. The dataset was randomly split into a training set (70%) and a validation set (30%).

**Results:**

A total of 12,858 eligible patients were included in our study, of whom 60.88% had a PloS. The GB classifier best predicted PloS (accuracy 75%, AUC 75.4%, Brier score 0.181), followed by LoR classifier (accuracy 75%, AUC 75.2%, Brier score 0.182). These models also showed to be adequately calibrated. Ridge and XGBoost regressions best predicted LoS, with the smallest total prediction error. The overall prediction error is between 6 and 7 days, meaning there is a 6–7 day mean difference between actual and predicted LoS.

**Conclusion:**

Our results demonstrate the potential of machine learning-based methods to predict LoS and provide valuable insights into the risks behind prolonged hospitalizations. In addition to physicians' clinical expertise, the results of these models can be utilized as input to make informed decisions, such as predicting hospitalizations and enhancing the overall performance of a public healthcare system.

## 1. Introduction

### 1.1. Importance of addressing hospitalization LoS after an emergency department visit

The Length of Stay (LoS) measures the time a patient spends in a hospital, from admission to discharge. It is a key indicator of the quality of hospital services, including the speed and efficiency of patient treatment, the prevention of hospital-acquired infections, the ability to anticipate prolonged stays due to pre-existing medical conditions, resource utilization, and the cost of inpatient care. LoS can also be used to evaluate the success of surgical procedures and patient outcomes. With an in-depth understanding of LoS and potential adverse events, hospitals can make informed decisions and improve patients' overall quality of care. Accurate LoS prediction enables the efficient use of medical resources, better clinical decision-making, and provision of useful prognostic information. In hospital management, LoS is critical in determining hospital costs and patient satisfaction. Furthermore, it is associated with disease severity and mortality (Paterson et al., 2006). During an ED visit, some predictors of hospital LoS were known before admission to the hospital. Prior studies have shown that patients in EDs have a longer LoS (Krochmal and Riley, 1994; Liew et al., 2003). It has been demonstrated that extended hospital stays negatively affect clinical outcomes: according to Sud et al. (2017), long LoS is associated with increased mortality and readmission rates; the results of Bo et al. (2016) indicated that PLoS is associated with cognitive impairment, functional limitations, and higher burdens of comorbidity; the results of Emori and Gaynes (1993) also indicated that PLoS increased the risk of hospital-acquired infections. Patients are prioritized based on their level of medical need in a triage plan to enhance healthcare and reduce mortality. Models that predict patient-related outcome measures and LoS are useful tools for maximizing healthcare utilization (Gellman, 1974). As a result, policymakers and clinicians could determine how to allocate resources among different approaches by comparing treatments across disciplines.

### 1.2. Methodological review/predictive modeling of PLoS

Machine learning (ML) provides innovative methods in data predictions that are widely used. Numerous studies have examined how different predictive models can predict LoS more accurately (Lu et al., 2015). A prediction model based on factors affecting LoS has been developed in previous studies using multiple supervised learning techniques. For categorical outcomes, including logistic regression (LoR), random forest (RF), Gradient Boosting (GB), K-nearest neighbors (KNN), support vector machine (SVM), decision tree (DT), and artificial neural networks (ANN; Hachesu et al., 2013; LaFaro et al., 2015) were used to predict LoS. In a study by Chuang et al. (2018), LoR, SVM, RF, multivariate adaptive regression splines (MARS), classification and regression tree (CART), etc. were used to study the prediction of PLoS in patients undergoing general surgery. The RF classifier showed the highest performance. In another interesting study, for a continuous outcome, Caetano et al. (2015) used and compared regressors, including multiple regression (MR), RF regression, decision tree (DT), neural network (NN), and support vector machine (SVM) regression. The RF regression showed the highest performance. The performance of ensemble learning models (like RF, GB, AdaBoost) is usually better than that of single learning models (Han et al., 2019). An alternative, data-driven approach to predictive analytics in emergency care is available through preprocessing, data mining, and machine learning techniques applied to big data stored in electronic health records (EHRs; Yu et al., 2018). In other clinical data from inpatients with lower limb fractures, Colella et al. (2021) employed similar ML techniques to predict PLoS, by dividing the outcome variable into two classes. Kirchebner et al. (2020) conducted an exploratory study on hospitalized schizophrenic patients to predict PLoS. This study selected the most significant features using a forward selection procedure. Then various machine learning classification algorithms were used for binary outcomes: with and without prolonged LoS. Overall in the literature, SVM, GB, LoR, NN, and RF are the most common and widely used supervised ML classifier algorithms used to estimate LoS (Jiang et al., 2010; Morton et al., 2014). Table 1 provides a brief overview of ML models, prediction outcomes, and the target groups for which LoS was predicted.

**Table 1:** Brief review of ML models and patients groups for predicting hospital patients' LoS.

**References**	**Outcome: prediction type**	**ML models**	**Target group**	**Results**
Mekhaldi et al. (2020)	Regression	RF Regressor, Gradient Boosting Regressor	General patients	GB performed better than RF; performance were checked by MSE, the R-squared and the Adjusted R-squared.
Daghistani et al. (2019)	Classification	RF, ANN, SVM, BN	Cardiac patients	RF model outperformed all other models: sensitivity (0.80), accuracy (0.80), and AUROC (0.94).
Tsai et al. (2016)	Regression	LR, ANN	Cardiac patients	LR model performed slightly better than ANN models, with a MAE value of 3.76 and 3.87
Symum and Zayas-Castro (2020)	Classification	DT C5.0, linear SVM, KNN, RF, and multi-layered artificial neural net	Chronic disease (congestive heart failure, acute myocardial infarction, COPD, pneumonia, type 2 diabetes).	For all patient groups, LSVM (Lagrangian SVM) with wrapper feature selection performed well.
Tanuja et al. (2011)	Classification	Naive Bayes; KNN; DT classifiers; Multi-layer backpropagation	General patients	MLP and NB models had the best classification accuracy of around 85%, while KNN performed poorly with only 63.6% accuracy
Combes et al. (2014)	Regression and classification	Two based models: *Classifier*: RF, LMT (Logistical model tree), MP, DT (C4.5-J48), NBTree, REPTree, and SVM. *Regression*: LR, SV regression, MLP, IRM (Isotonic regression model), M5P, PRLM (Pace regression linear models)	Pediatric	Using 10-fold cross-validation, obtained the best performances in using logistic regression, and in continuous outcome SVM Regression showed a lower prediction error.
Etu et al. (2022)	Classification	LoR, GB, DT, and RF	COVID-19 Patients	The GB model outperformed the baseline classifier (LoR) and tree-based classifiers (DT and RF) with an accuracy of 85% and F1-score of 0.88 for predicting ED LoS
Alsinglawi et al. (2020a)	Regression	RF Regressor; GB Regressor; Stacking Regressor; DNN	Cardiovascular patients in the ICU	GB regressor outweighed the other proposed models, and showed a higher R-squared.
Kirchebner et al. (2020)	Classification	BT; KNN; SVM	Schizophrenic patients	Two factors have been identified as particularly influential for a prolonged forensic LoS, namely (attempted) homicide and the extent of the victim's injuries.
Thongpeth et al. (2021)	Regression	LR with three penalized linear (ridge, lasso, elastic net), and 4 ML model types: SVR, NN, RF, and XGBoost	Chronic disease	The RF model had the best predictive performance with the smallest prediction errors, while linear ridge regression had the poorest prediction performance with the largest prediction errors.

LoR, logistic regression; LR, linear regression; RF, random forest; NB, Naive Bayes; ANN, artificial neural network; SVM, support vector machine; MLP, Multi-layer backpropagation; DT, decision tree; GB, Gradient Boosting; XGBoost, eXtreme Gradient Boosting; KNN, K-nearest neighbors; BN, Bayesian network; COPD, chronic obstructive pulmonary disease; MSE, mean square error; ICU, intensive care unit.

### 1.3. Related works

Previous research has investigated various methods of predicting LoS with varying scopes and settings. LoS can be predicted for all patients admitted to the hospital based on non-medical factors such as type of admission, gender, race, insurance status, place of residence, and the cost of hospitalization, as well as medical characteristics like risk/severity measures, primary condition groups, emergency degree, and prior admissions. It is also possible to predict LoS for specific diseases or surgical procedures. The most frequently reported factors that affect the ED LoS are patient age, gender, triage category, mode of arrival, the requirement for an interpreter, admission, diagnostic complexity necessitating extra testing, and the availability of resources, including staff and beds (Asaro et al., 2007; Biber et al., 2013; Rahman et al., 2020). Patient characteristics influencing LoS, such as demographics and comorbidities, are often available at triage and admission (Tsai et al., 2016). Several studies in the literature have examined the LoS trends in general patients (Tanuja et al., 2011; Mekhaldi et al., 2020), or in particular patient populations, focusing, for instance, on a certain age group (Ackroyd-Stolarz et al., 2011; Launay et al., 2018; Marfil-Garza et al., 2018; Sir et al., 2019) or specific health conditions (e.g., cardiology; García-González et al., 2014; Tsai et al., 2016; Chuang et al., 2018; Daghistani et al., 2019), peritoneal dialysis (Wu et al., 2020), schizophrenia (Kirchebner et al., 2020), knee arthroplasty (Song et al., 2020), COVID-19 (Vekaria et al., 2021; Etu et al., 2022; Zeleke et al., 2022), abdominal pain (Dadeh and Phunyanantakorn, 2020), mental health (Wolff et al., 2015), cardiovascular diseases (Almashrafi et al., 2016; Alsinglawi et al., 2020a), or in specific discipline areas or specialties such as spine surgery (Basil and Wang, 2019) and cancer surgeries (Laky et al., 2010; Gohil et al., 2014; Jo et al., 2021). However, most of these studies have had limited sample sizes and have not considered a wide range of clinical factors. In-hospital adverse events are known to increase the risk of prolonged Length of Stay (LoS) in older patients (Ackroyd-Stolarz et al., 2011).

A study of Length of Stay (LoS) in the emergency department of a tertiary care center (van der Veen et al., 2018) found a significant association between multiple chief complaints, including headaches and chest pain, laboratory/radiology testing, and consultation with prolonged hospitalization in the ED. Another population-based study conducted in Osaka, Japan (Katayama et al., 2021) showed that factors such as old age, traffic accidents, lack of a permanent address, need for nursing care, and being solitary were associated with prolonged hospitalization for patients transported by ambulance. Another retrospective study of prolonged LoS in a tertiary healthcare center in Mexico (Marfil-Garza et al., 2018) showed that demographic and disease-specific differences, such as younger age, male gender, lower physician-to-patient ratio, emergency and weekend admissions, surgery, number of comorbidities, and lower socioeconomic status, were associated with a prolonged LoS. Diseases with the greatest risk for prolonged LoS included complex conditions like bone marrow transplant, systemic mycoses, parasitosis, and complex abdominal diseases like intestinal fistulas.

### 1.4. Aims

This study used various supervised machine learning algorithms to predict the length of stay for patients admitted through the emergency department in general patient settings. The outcome was analyzed as both a dichotomous (PLoS) and continuous (LoS) variable. Data was gathered from routine triage and ED admission processes and recorded in the hospital's electronic medical records. The best-performing model was selected to make predictions and gain meaningful insights for future patients.

## 2. Materials and methods

### 2.1. Study design and population

**Figure 1:**
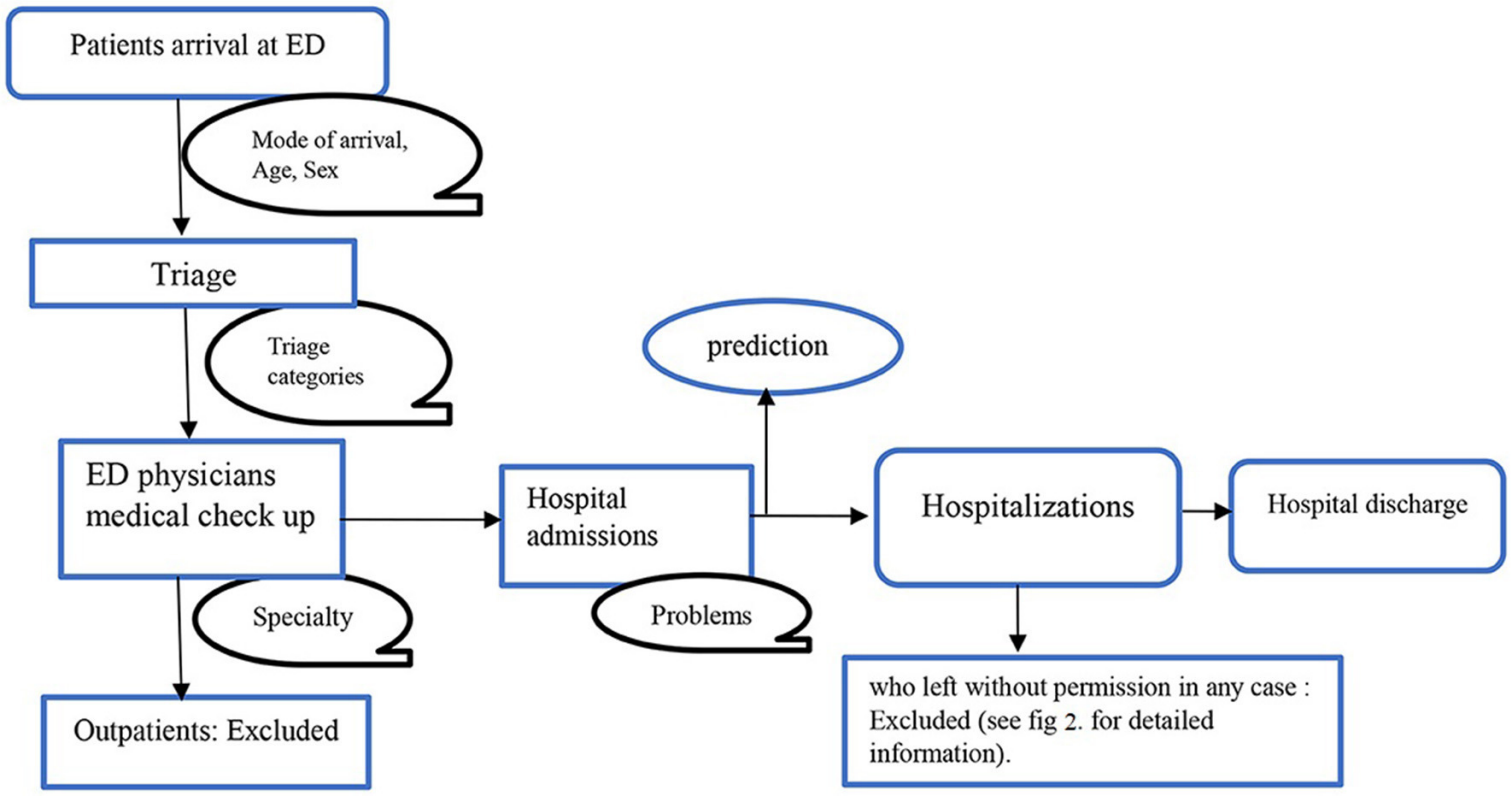
Clinical pathway.

**Figure 2:**
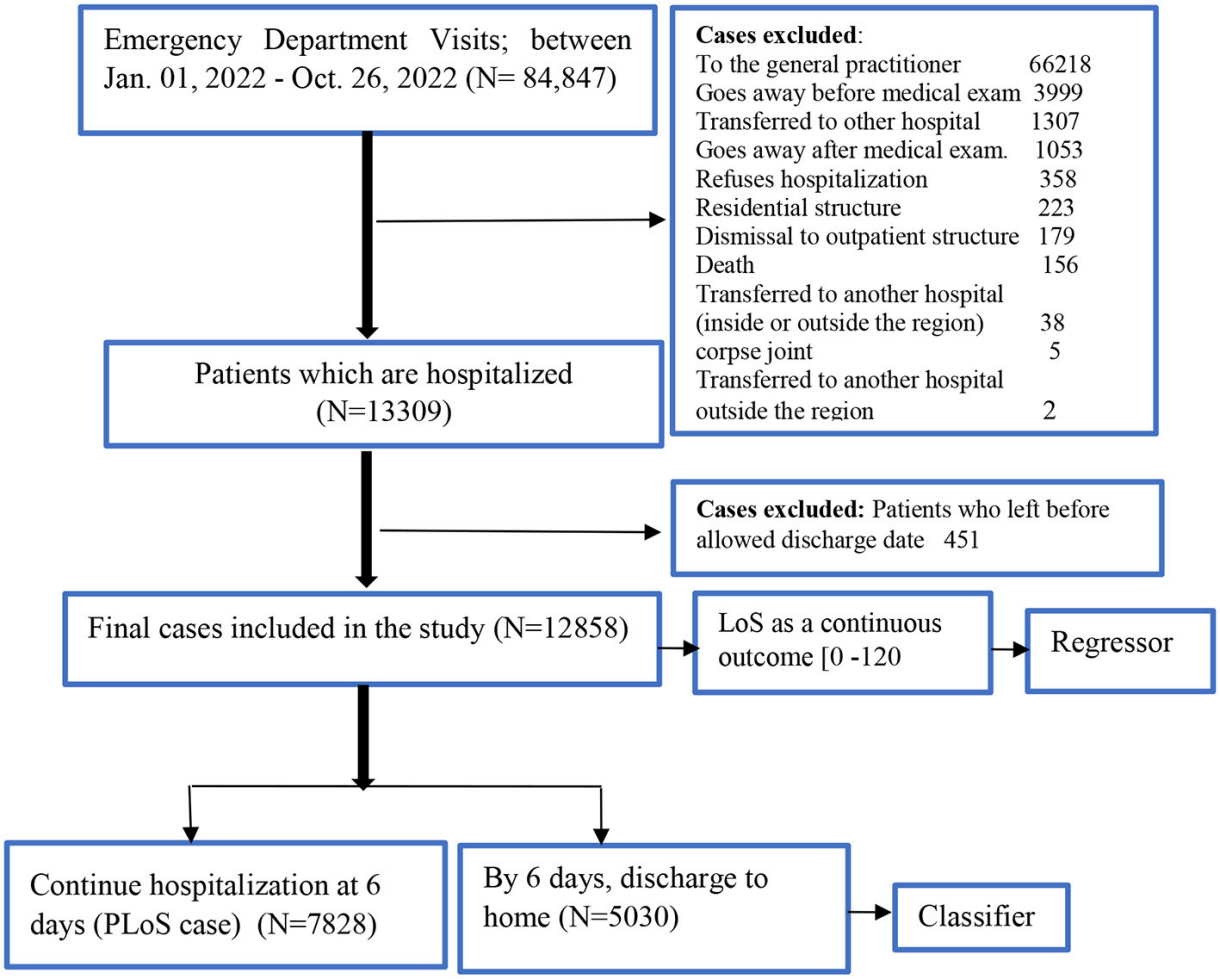
Flowchart of patients selection.

We screened for eligibility for all admissions to the hospital through the ED of the public University Hospital of Bologna Sant'Orsola-Malpighi (AOSP), Bologna, Italy, between January 1, 2022, and October 26, 2022. AOSP is a 1,500-bed tertiary care teaching hospital in Central-Northern Italy with 70,000 emergency department visits per year, this is one of the largest hospitals in the country (Fridman et al., 2022). All the necessary steps of the clinical pathway: ED triage, medical examination, hospital admission, and hospital discharge, are shown in Figure 1. We included all patients who visited the ED, were admitted to the hospital, and stayed until they got formal permission to discharge. Any patients who left the ED, were transferred to another hospital, refused the hospitalization, died, went away after the medical examination, left without being seen, or left without notice (detail as shown in Figure 2) were excluded from the analysis.

#### 2.1.1. Outcome variable

The primary outcome of this study was hospital length of stay (LoS) and prolonged length of stay (PloS). LoS is calculated as the number of days between admission and discharge. We defined PLoS threshold as any LoS that is longer than the reported average LoS (i.e., 6 days; Zoller et al., 2014; Song et al., 2020; Wu et al., 2020). The LoS was reclassified as binary (i.e., either “without PLoS < 6 ‘days' or with PLoS” ≥6 “days”) for classification analysis, and LoS as a continuous outcome for regression analysis.

#### 2.1.2. Independent variables

Any information collected at triage and available from ED admissions was considered as a predictor of LoS or PLoS. These include demographic factors (such as gender and age), mode of arrival/source of admission, risk categories as determined by triage at the entrance, and current problems or chief complaints. A detailed description of each independent feature, measure category, and outcome is presented in Supplementary Table 1.

### 2.2. Model development

#### 2.2.1. Predictive models fitting and evaluation: binary outcome

**Figure 3:**
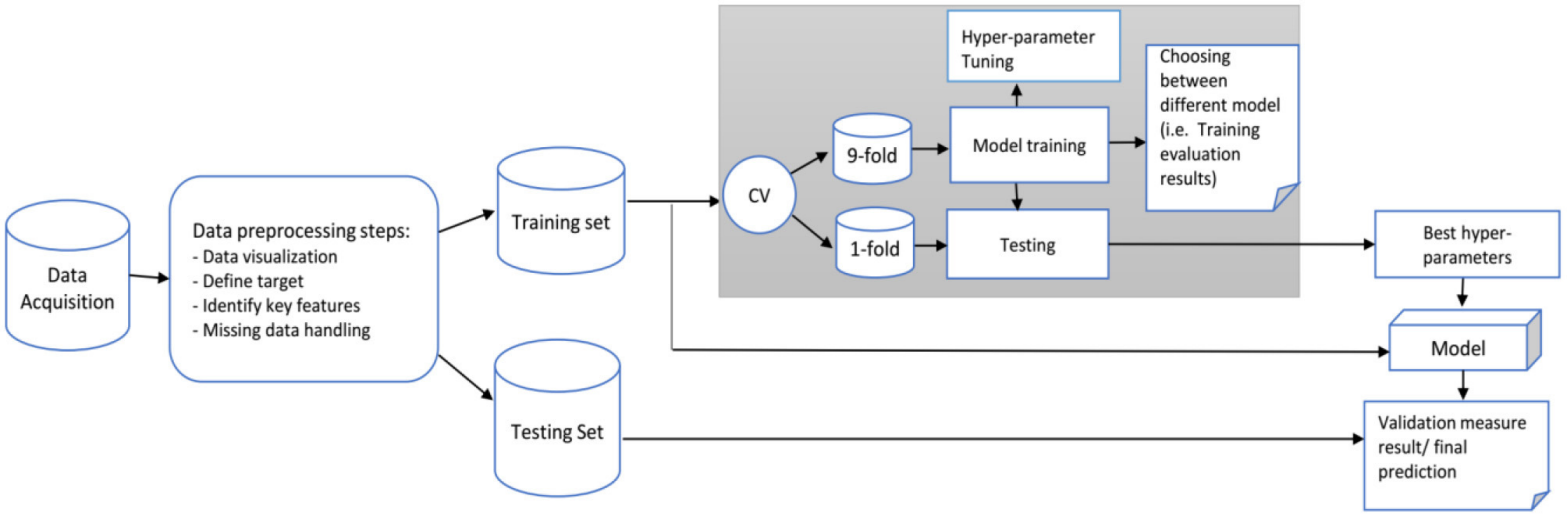
Proposed framework for our prediction model.

The diagram in Figure 3 shows the data analysis framework we followed for developing and evaluating our predictive model. The main objective is to predict the categorical class labels of new data points or instances based on past observations. Based on the literature, six common classification algorithms were selected for comparison: GradientBoosting (GB), random forests (RF), support vector machine (SVM), K-Nearest Neighbors (KNN), AdaBoost, and logistic regression (LoR). The model with the highest prediction performance was used to identify predictive factors contributing to the outcome. We randomly divided the data into training (70%) and testing or validation (30%) sets. The analyses were performed in *Scikit-learn* in Python (Jupyter notebook version; Pedregosa et al., 2011).

Hastie et al. (2009) provide detailed explanations, but here we provide a brief overview of ML techniques, and hyperparameters tuning settings.

##### 2.2.1.1. Random Forests (RF)

In statistical applications, Random Forests (RF) are a commonly used type of supervised machine learning that can be utilized for both classification and regression tasks (Breiman, 2001; Genuer et al., 2010). RF predicts outcome labels for a group of samples by building several decision trees using a random set of covariates. The weak classifier can be transformed into a strong one by taking the majority of votes for classification and averaging in regression. To enhance the classification accuracy, multiple decision trees are combined in RF to form an ensemble classification algorithm. Each tree is grown using a bootstrapped sample from the original data (Qi, 2012). An ensemble ML method combines a series of underperforming classifiers to produce an improved classifier. The mechanism for this combination differs between ensemble algorithms. In this study, the RF model was created using the *sklearn.RandomForestClassifier* package in Python (Pedregosa et al., 2011).

##### 2.2.1.2. Gradient Boosting (GB)

Gradient Boosting is an ensemble learning model that employs decision trees as its base classifier, without bootstrap sampling (Luo et al., 2020). GB aims to create a robust predictive model by combining weak learning models, considering the bias of all previous decision trees in the model. Furthermore, unlike randomization in other methods, GB focuses on fixing the target outcomes in order to minimize errors. In this study, the GB model was constructed using the *sklearn.GradientBoostingClassifier* package in Python (Pedregosa et al., 2011).

##### 2.2.1.3. Support vector machines (SVMs)

In SVMs, the data is separated using a large gap or hyperplane to deal with linearly non-separable problems. It works by finding an optimal separating hyperplane in the feature space for classification. The Python *sklearn.SVC* package was used to build the SVM model for this study (Pedregosa et al., 2011).

##### 2.2.1.4. AdaBoost classifier

Similar to GB, AdaBoost classifier is also a boosting algorithm, converting a set of weak learners into a single strong learner. However, they differ on how they create weak learners during the iterative process. In GB, as mentioned, it is to minimize the cumulative predicted errors. Still, in AdaBoost it focuses on training the prior miscalculated observations and alters the data distribution to improve sample weight values. The Python *sklearn.AdaBoostingClassifier* package was used to build the AdaBoost model for this study (Pedregosa et al., 2011).

##### 2.2.1.5. K-Nearest Neighbors (KNN)

KNN is an instance-based algorithm, which labels the test record based on its distance from similar data during training (i.e., which analyzes the similarities between the new data and the existing data and adds the new data into the category that is highly similar to the available categories). The only step in building the model is storing the training dataset. Then, the algorithm finds the closest data points in the training dataset, or its “nearest neighbors” to predict a new data point (Keller et al., 1985). Python *sklearn. KNeighborsClassifier* package was used to build the AdaBoost model for this study (Pedregosa et al., 2011).

##### 2.2.1.6. Logistic regression (LoR)

The LoR model is widely used in binary classification problems. The parameter of interest is estimated using maximum likelihood estimation. Similarly, Python *sklearn.LogisticRegression* package was used for this classifier.

Every machine learning (ML) technique requires the optimization of hyperparameters to enhance its performance. To develop a well-performing generalized model, it is crucial to carefully select the hyperparameters. Different algorithms will have distinct sets of hyperparameters.

The hyperparameter tuning summary for each type of classifier and their descriptions used for this analysis are shown in Table 2.

**Table 2:** Hyperparameter tuning summary.

**Model classifiers**	**Hyperparameter tuning description**
RF	# of _estimators = 200; longest path between root node and leaf node, max_ depth = 15; class_ weight = “balanced;” Number of maximum features for each tree, max_ features = sqrt; min_ samples_ split = 2; min_ samples_ leaf = 1; random_ state = 42
GB	# of estimators = 200, max_depth = 4, and loss = ls
KNN	Number of neighbors = 10; algorithms = “auto;” leaf_ size = 1; *p* = 1; weights = “uniform”
AdaBoost	Similar to RF, define the Decision tree (Dt) classifier first in the same setting and then boost the Dt fit by AdaBoostClassifier.
SVM	Kernel = linear; degree of similarity, gamma = 0.01; regularization, C = 10
LoR	No critical hyperparameters need to be tuned.

In building a prediction model, evaluating its performance and accuracy is important. Various metrics were used to assess the model's accuracy, including the Brier score, AUC, accuracy, sensitivity, specificity, precision, and F1-measure (Steyerberg et al., 2010). Calibration curve plots were also employed to visualize the calibration power of each model and ensure that the model fitted the data optimally. By carefully evaluating the predictive power of the model, we can ensure that the results produced by the model are reliable and can be trusted for decision-making purposes in the healthcare system.

Brier score is an overall performance measure, a measure of the accuracy of a predicted probability score (i.e., mean squared error of probability estimate). A low Brier score suggests an excellent overall performance (Steyerberg et al., 2010).


$$\begin{array}{c} BS=\frac{\underset{i=1}{\sum }{\left(\hat{p}\left(y_{i}\right)-y_{i}\right)}^{2}}{n} \end{array}$$

An evaluation metric like accuracy calculates the proportion of correct predictions (both positive and negative) out of all the predictions made by the model. Achieving the highest accuracy level is important. Sensitivity or recall reflects the number of positive predictions that were accurately identified, while specificity measures the same for negative predictions. A higher recall indicates that more true values were correctly predicted. The F1-score balances precision and recall by taking the harmonic mean of both values. The overall predictive accuracy of the model was evaluated by determining the area under the receiver operating characteristic curve (AUC). Calibration is crucial in developing and validating clinical prediction models, which refers to the match between predicted and observed risks (Steyerberg, 2019). In the case of binary outcomes, calibration measures the agreement between estimated and observed probabilities of occurrence. Calibration curves were used to assess calibration. A perfect model's calibration curve would be diagonal, meaning that the predicted probabilities align with the observed probabilities.

##### 2.2.1.7. Variables importance

The most effective prediction model was utilized to determine the importance of variables. Identifying key factors in machine learning predictions is crucial. The metric used to evaluate this is the mean decrease in impurity, which calculates the average change in the impurity of nodes across all trees in the ensemble, taking into account the proportion of samples that reach each node. A higher value generally means that the feature is more significant. With high-dimensional datasets, it is crucial to properly select and rank covariates for both prediction and interpretation purposes.

#### 2.2.2. Predictive models fitting and evaluation: continuous outcome

In order to minimize information loss in a classification task, we also explored it as a continuous outcome and employed regression models. Our study employed eight different learning algorithms, including linear regression (LR) and its penalized versions (Lasso, Ridge, and Elastic Net regression), as well as Support Vector Regression, Random Forest Regression, K-Nearest Neighbors (KNN), and XGBoost Regression.

##### 2.2.2.1. Linear regression (LR)

This method involves fitting a linear equation to the data to establish a relationship between the independent variables and the dependent or outcome variable. The equation can then be used to make predictions based on the input data. The linear regression model is typically expressed in the following form:


$$\begin{array}{c} y_{i}=\beta_{0}+\overset{n}{\underset{j=1}{\sum }}\beta_{j}x_{ij} \end{array}$$

where *y*_*i*_ is the continuous outcome value of subject i, β_0_ is intercept, β_*j*_ is the coefficient of feature j, and *x*_*ij*_ is feature j of subject i.

It is possible to estimate the regression parameter of a linear regression model using the least square method by minimizing the error term in the unknown β_*j*_.


$$\begin{array}{c} \hat{\beta }=argmin_{\beta }\left\{\frac{1}{n}\overset{n}{\underset{i=1}{\sum }}{\left(y_{i}-{ŷ}_{i}\right)}^{2}\right\} \end{array}$$

##### 2.2.2.2. Ridge regression

It works by finding the coefficients that minimize the sum of error squares by applying a penalty to those coefficients (Tibshirani, 1996).


$$\begin{array}{c} \hat{\beta }=argmin_{\beta }\left\{\frac{1}{n}\overset{n}{\underset{i=1}{\sum }}{\left(y_{i}-{ŷ}_{i}\right)}^{2}+\lambda \overset{p}{\underset{j=1}{\sum }}{\beta_{j}}^{2}\right\} \end{array}$$

λ is the regularization parameter that we are going to optimize.

##### 2.2.2.3. Lasso regression

The same task but uses the sum of absolute values of the weights for the penalty (Tibshirani, 1996).


$$\begin{array}{c} \hat{\beta }=argmin_{\beta }\left\{\frac{1}{n}\overset{n}{\underset{i=1}{\sum }}{\left(y_{i}-{ŷ}_{i}\right)}^{2}+\lambda \overset{p}{\underset{j=1}{\sum }}|\beta_{j}|\right\}\; \end{array}$$

##### 2.2.2.4. Elastic-Net

A combination of lasso and ridge regression that reduces bias, better than lasso or ridge regressions (Friedman et al., 2009).


$$\begin{array}{c} \hat{\beta }=argmin_{\beta }\left\{\frac{1}{n}\overset{n}{\underset{i=1}{\sum }}{\left(y_{i}-{ŷ}_{i}\right)}^{2}+\lambda_{1}\overset{p}{\underset{j=1}{\sum }}{\beta_{j}}^{2}+\lambda_{2}\overset{p}{\underset{j=1}{\sum }}|\beta_{j}|\right\} \end{array}$$

In contrast to prediction models, regression models focus on estimating the relationship between a set of independent variables and a continuous outcome variable. Instead of categorizing the outcome into specific classes, the regression models aim to predict the continuous value of the outcome based on the given set of independent variables. The performance measure used in regression models is typically the mean squared error, or the root mean squared error, which represents the average deviation between the predicted and actual values of the outcome variable. Regression models aim to minimize these errors, thereby providing a more accurate prediction of the continuous outcome.

Using a loss function helps us evaluate the performance of a prediction model by quantifying the difference between the predicted and the actual values. Mean square error (MSE), mean absolute error (MAE), and mean relative error (MRE) were calculated to measure the prediction performance of each model. MSE is the most widely used loss function for continuous outcomes. Still, we also considered MAE and MRE to get a more comprehensive understanding of the performance. The lower the value of the loss function, the better the model's prediction performance.

$MSE=\frac{{\overset{n}{\underset{i=1}{\sum }}\left({ŷ}_{i}-y_{i}\right)}^{2}}{n}$; $MAE=\frac{\overset{n}{\underset{i=1}{\sum }}|{ŷ}_{i}-y_{i}|}{n}$; and $MRE=\frac{\overset{n}{\underset{i=1}{\sum }}\left(\frac{\left|{ŷ}_{i}-y_{i}\right|}{y_{i}}\right)}{n}$ where $\hat{y_{i}}$ and *y*_*i*_ are the predicted LoS and actual LoS for the *i*th test data.

## 3. Results

### 3.1. Patient selection

Figure 1 illustrates a flowchart of patients' eligibility for analysis in the emergency department of triaging system. A total of 84,847 patient visits were recorded at the ED between January 1 and October 26, 2022. After filtering for exclusion criteria, 12,858 patients were available for analysis.

### 3.2. Descriptive statistics

#### 3.2.1. Patients characteristics summary

Of the 12,858 eligible patients included in the study, 60.88% had a prolonged length of stay (LoS). The median age of the patients was 72 years, and the elderly age groups (50–69 and 70+) had longer LoS than the other age groups. The male patients comprised 52.6% (6,757/12,858) of the total population. 51.7% of the patients arrived at the hospital via ambulance and had a longer stay compared to those who arrived by car or on foot. In the triage categories, patients with red codes, which indicate an higher severity at the ED admission, had a longer LoS, while green and white codes showed shorter stays. Light blue codes were also associated with prolonged LoS.

**Figure 4:**
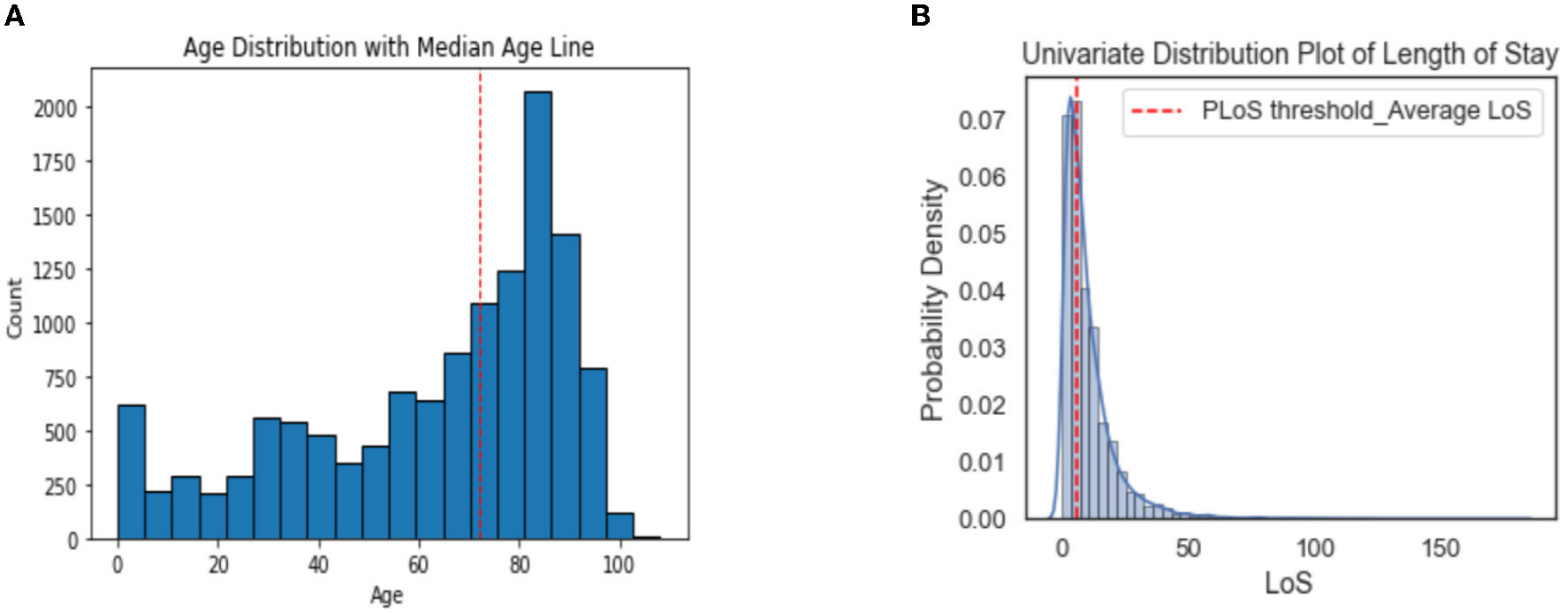
Histograms showing the distribution of Age **(A)** and LoS **(B)** in all patients.

**Figure 5:**
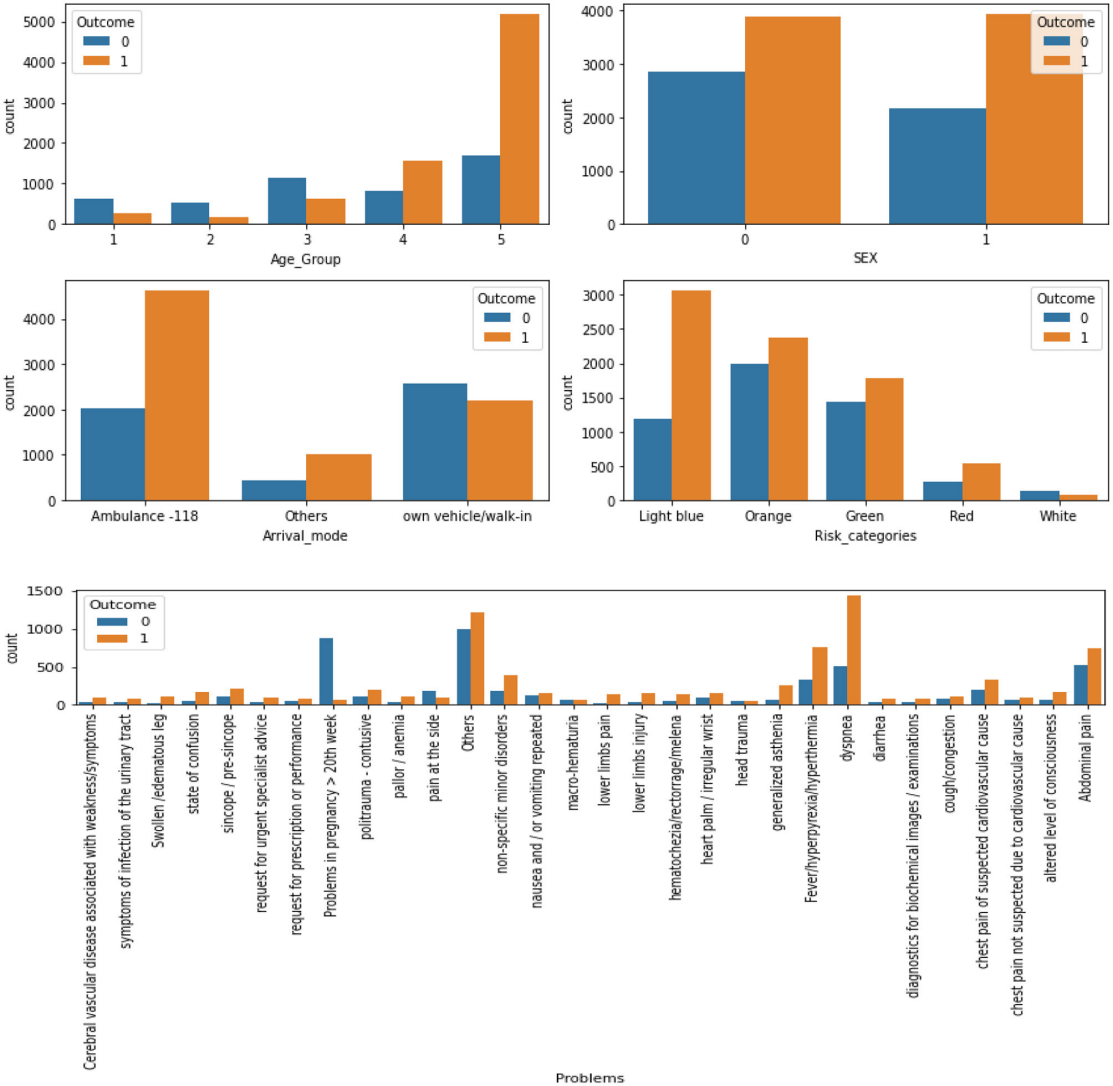
The results for each factor's dichotomous outcome (0, LoS < 6, without PLoS; 1, LoS ≥ 6, with PLoS).

**Figure 6:**
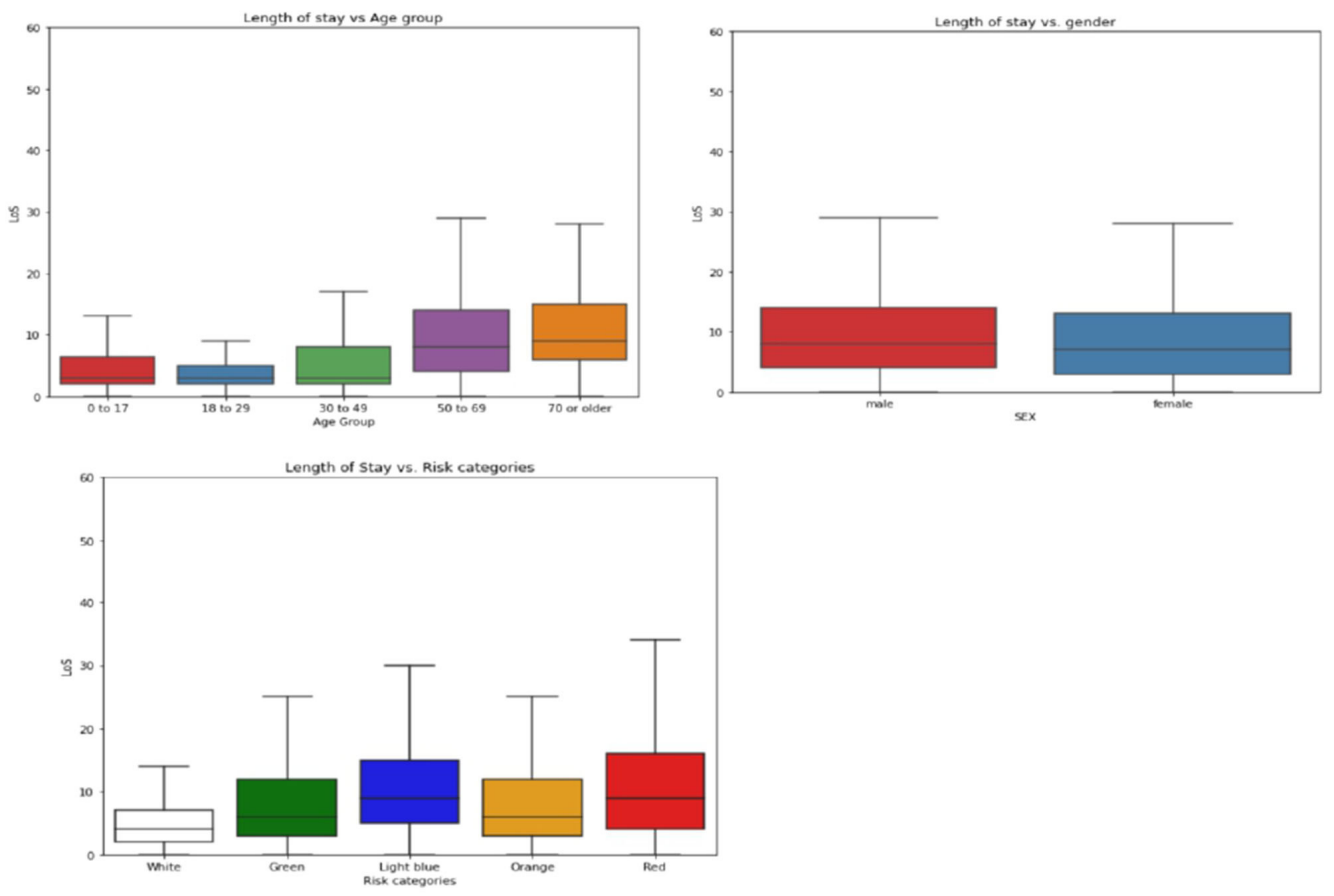
Boxplots of length of stay (LoS) on demographic factors, separated into two panels. **(Upper panel)** Shows LoS boxplots for age groups **(left)** and sex **(right)**. **(Lower panel)** Shows the box plot for risk categories of triage evaluations.

The most common problems among the patients were dyspnea (15.2%), abdominal pain (9.9%), and fever/hyperpyrexia/hyperthermia (8.5%). The majority of patients were seen by specialists in general medicine (29.2%), geriatrics (12.6%), astanteria or casualty department (10.7%), obstetrics and gynecology (9.0%), and pediatrics (4.7%). A detailed breakdown of patient characteristics can be found in Table 3. The count plots for each patient for each specialty and problems are included in the Appendix, in Supplementary Figures 1, 2, respectively.The distribution of length of stay (LoS) for the patients is depicted in a histogram in Figure 4. The distribution of LoS values was found to be right-skewed, with a majority of patients having an LoS ranging from 1 to 20 days. To further explore the impact of different factors on LoS, a visualization of the dichotomous outcome result for each factor is presented in Figure 5, while Figure 6 shows the continuous outcome for each factor. By examining these visualizations, we can gain insights into which factors may significantly impact LoS and further investigate the relationships between these factors and patient outcomes. Overall, these figures provide a clear and concise way to understand the distribution of LoS values and their relationship with different factors.

**Table 3:** Presenting characteristics of patients who visited the ED of ASOP, Bologna, Italy, 2022 (*n* = 12,858).

**Factor**		**PLoS (i.e.**, ≥**6 days)**		
	**Total**, ***n*** **(%) (*****n*** = **12,858)**	**With PLoS (*****n*** = **7,828) 60.88%**	**Without PLoS (*****n*** = **5,030) 39.12%**	**Proportion difference (%) (with PLoS—without PLoS)**
Age, median	72	**-**	**-**	**-**
**Age categories**, ***n*** **(%)**				
(0–17)	1,170 (9.1)	329 (4.2)	841 (16.7)	−12.5
(18–29)	679 (5.3)	158 (2.1)	521 (10.4)	−8.3
(30–49)	1,772 (13.8)	616 (7.9)	1,176 (23.4)	−15.5
(50–69)	2,364 (18.4)	1,554 (19.9)	810 (16.1)	3.8
70 or older	6,873 (53.5)	5,171 (66.1)	1,702 (33.9)	32.2
**Gender**, ***n*** **(%)**				
Male	6,101 (47.4)	3,928 (50.2)	2,173 (43.2)	7.0
Female	6,757 (52.6)	3,900 (49.8)	2,857 (56.8)	−7.0
**Mode of arrival**, ***n*** **(%)**				
Ambulance–118	6,645 (51.7)	4,624 (59.1)	2,021 (40.2)	18.9
Own vehicle/walk-in	4,769 (37.2)	2,204 (28.2)	2,565 (51.0)	−22.8
Others^a^	1,444 (11.2)	1,000 (12.8)	444 (8.8)	4.0
**Triage category**				
Red	807 (6.3)	539 (6.9)	268 (5.3)	1.6
Orange	4,360 (33.9)	2,367 (30.2)	1,993 (39.6)	−9.4
Light blue	4,253 (33.1)	3,065 (39.2)	1,188 (23.6)	15.6
Green	3,224 (25.1)	1,784 (22.8)	1,440 (28.6)	−5.8
White	214 (1.7)	73 (0.9)	141 (2.8)	−1.9
**Specialty**, ***n*** **(%)**				
General medicine	3,757 (29.2)	2,995 (38.3)	762 (15.1)	23.2
Geriatric	1,624 (12.6)	1,252 (16.0)	372 (7.4)	8.6
Astanteria/casualty department	1,450 (10.7)	809 (10.3)	641 (12.7)	−2.4
Obstetrics and gynecology	1,159 (9.0)	114 (1.5)	1,045 (20.8)	−19.3
Pediatrics	609 (4.7)	193 (2.5)	416 (8.3)	−5.8
General surgery	571 (4.4)	276 (3.5)	295 (5.9)	−2.4
Infectious and tropical diseases	533 (4.1)	372 (4.8)	161 (3.2)	1.6
Orthopedics and traumatology	481 (3.7)	378 (4.8)	103 (2.0)	2.8
Urology	405 (3.2)	99 (1.3)	306 (6.1)	−4.8
Coronary unit	377 (2.9)	283 (3.6)	94 (1.9)	1.7
Pediatric surgery	376 (2.9)	77 (1.0)	299 (5.9)	−4.9
Gastroenterology	308 (2.4)	237 (3.0)	71 (1.4)	1.6
Cardiology	150 (1.2)	96 (1.2)	54 (1.1)	0.1
Intensive care	141 (1.1)	113 (1.4)	28 (0.6)	0.8
Pneumology	135 (1.1)	111 (1.4)	24 (0.5)	0.9
Nephrology	105 (0.8)	91 (1.2)	14 (0.3)	0.9
Oncology	93 (0.7)	67 (0.9)	26 (0.5)	0.4
Vascular surgery	89 (0.7)	65 (0.8)	24 (0.5)	0.3
Missing values	76 (0.6)	30 (0.4)	46 (0.9)	−0.5
Others^b^	419 (3.3)	170 (2.2)	249 (5.0)	−2.8
**Problems**, ***n*** **(%)**				
Dyspnea	1,954 (15.2)	1,446 (18.5)	508 (10.1)	8.4
Abdominal pain	1,268 (9.9)	739 (9.4)	529 (10.5)	−1.1
Fever/hyperpyrexia/hyperthermia	1,090 (8.5)	761 (9.7)	329 (6.5)	3.2
Problems in pregnancy > 20th week	944 (7.3)	70 (0.9)	847 (16.8)	−15.9
Non-specific minor disorders	579 (4.5)	395 (5.0)	184 (3.7)	1.4
Chest pain of suspected cardiovascular cause	524 (4.1)	329 (4.2)	195 (3.9)	0.3
Sincope/pre-sincope	344 (2.7)	220 (2.8)	114 (2.3)	0.5
Generalized asthenia	325 (2.5)	257 (3.3)	68 (1.4)	1.9
Politrauma—contusive	301 (2.3)	198 (2.5)	103 (2.0)	0.5
Pain at the side	278 (2.2)	100 (1.3)	178 (3.5)	−2.3
Nausea and/or vomiting repeated	269 (2.1)	150 (1.9)	119 (2.4)	−0.5
Heart palm/irregular wrist	251 (2.0)	156 (2.0)	95 (1.9)	0.1
Altered level of consciousness	234 (1.8)	165 (2.1)	69 (1.4)	0.7
State of confusion	213 (1.7)	162 (2.1)	51 (1.0)	1.1
Hematochezia/rectorrage/melena	194 (1.5)	136 (1.7)	58 (1.2)	0.6
Lower limbs injury	187 (1.5)	157 (2.0)	30 (0.6)	1.4
Cough/congestion	181 (1.4)	105 (1.3)	76 (1.5)	−0.2
Lower limbs pain	160 (1.2)	137 (1.8)	23 (0.5)	1.3
Chest pain not suspected due to cardiovascular cause	158 (1.2)	92 (1.2)	66 (1.3)	−0.1
Pallor/anemia	137 (1.1)	108 (1.4)	29 (0.6)	0.8
Request for urgent specialist advice	135 (1.0)	94 (1.2)	41 (0.8)	0.4
Macro-hematuria	130 (1.0)	70 (0.9)	60 (1.2)	−0.3
Diarrhea	121 (0.9)	85 (1.1)	36 (0.7)	0.4
Request for prescription or performance	120 (0.9)	75 (1.0)	45 (0.9)	0.1
Swollen/edematous leg	119 (0.9)	104 (1.3)	15 (0.3)	1.0
Weakness of extremities/symptoms associated with cerebrovascular disease	118 (0.9)	89 (1.1)	29 (0.6)	0.6
Symptoms of infection of the urinary tract	115 (0.9)	78 (1.1)	37 (0.7)	0.3
Diagnostics for biochemical images/examinations	108 (0.8)	77 (1.1)	31 (0.6)	0.4
Head trauma	99 (0.8)	54 (0.7)	45 (0.9)	−0.2
Other^c^	2,212 (17.2)	1,219 (15.6)	993 (19.7)	−4.2

^a^Taxi, helicopter 118, army ambulances, fire brigade, police, etc.

^b^Damages, Ent (ear, nose, and throat) problem, nephrology (enabled for transplantation), neonatology, pediatric oncology, semi-intensive therapy, maxillo facial surgery, hematology, thoracic surgery ophthalmology, heart surgery, neonatal intensive care, pediatric heart surgery, and dermatology.

^c^More than 135 cases.

**Figure 7:**
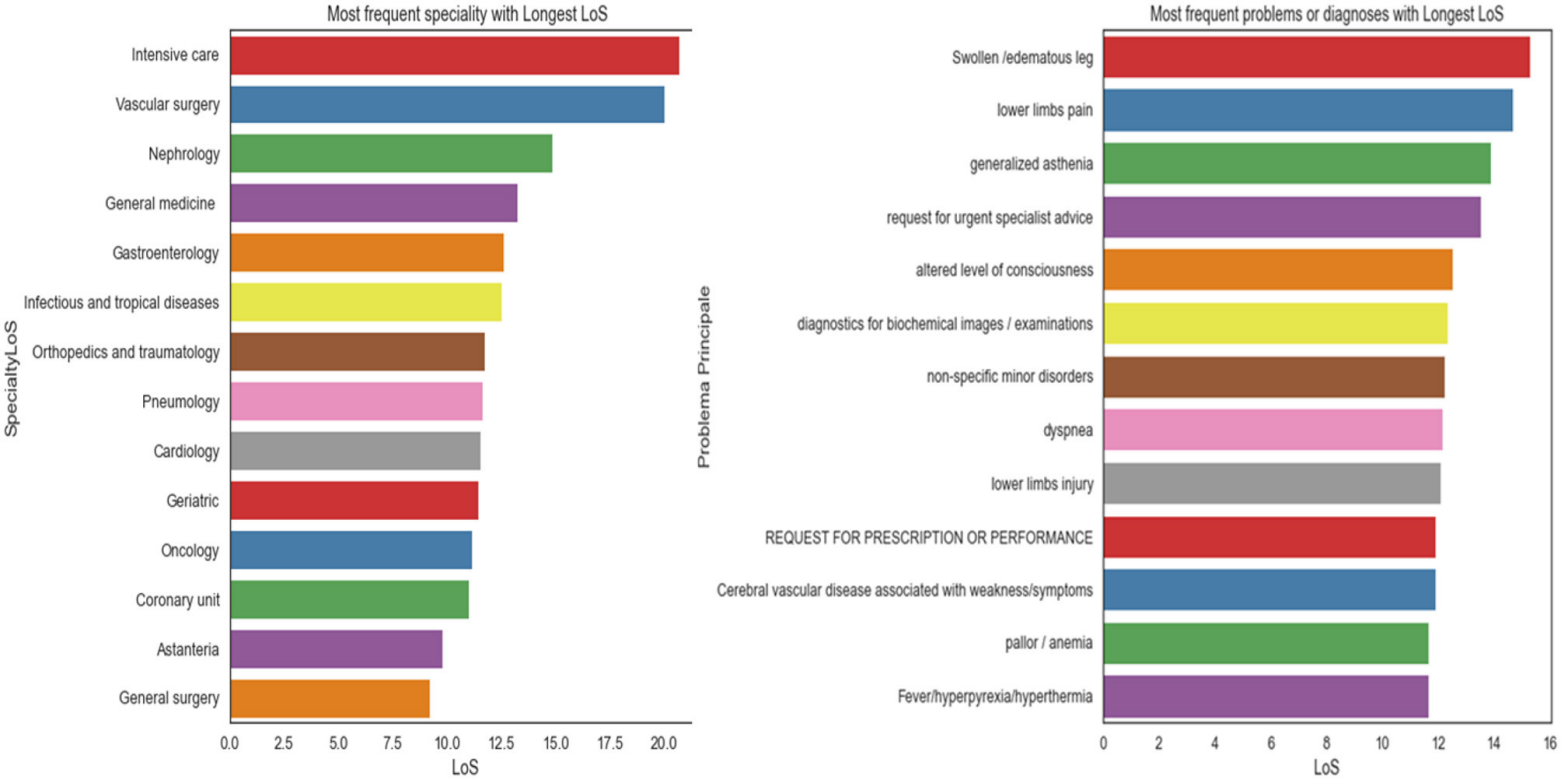
The average LoS for each problem **(right)** and for each specialty **(left)**.

Figure 7 displays the average LoS for each problem and specialty. The highest average LoS was observed in Intensive Care, Vascular Surgery, Nephrology, General Medicine, Gastroenterology, Infectious Diseases, Orthopedics and Traumatology, Pneumology, Geriatrics, Cardiology, Oncology, and the Coronary Unit, respectively. The average LoS was also higher for patients experiencing issues such as swollen/edematous legs, lower limb pain, generalized weakness, requests for urgent specialist advice, altered levels of consciousness, diagnostic tests for biochemical exams or images, non-specific minor disorders, dyspnea, lower limb injuries, requests for prescription refills, and pallor/anemia.

### 3.3. Prediction and model performance results: binary outcome

**Figure 8:**
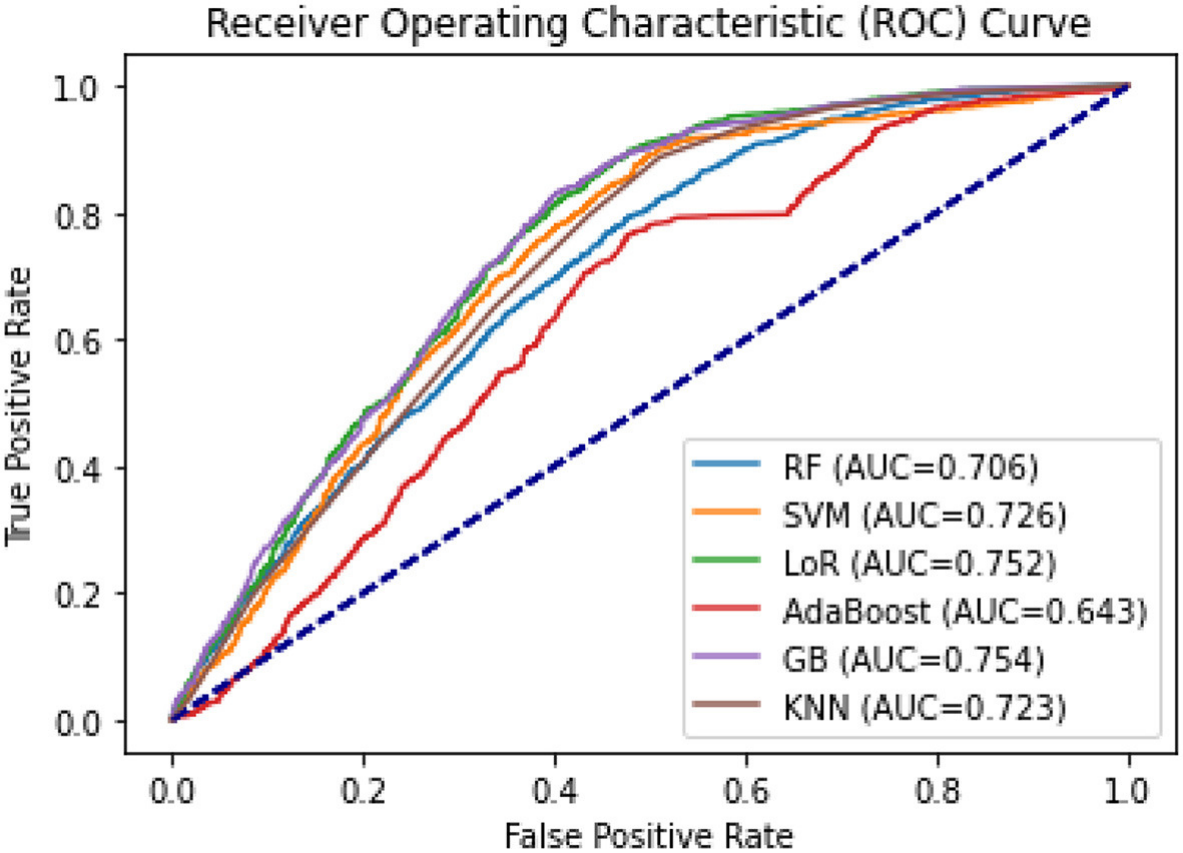
ROC curves and AUC of the six classification models for PLoS prediction.

The AUCs for all machine learning methods ranged from 0.643 for AdaBoost to 0.754 for GB (see Figure 8). GB was the best-performing classifier, followed by LoR (AUC = 0.752) and SVM (AUC = 0.726). The F1-scores ranged from 0.65 (AdaBoost) to 0.73 in GB, and 0.74 in LoR (see Table 4), indicating a high capability of these models to predict the prolonged length of stay.

**Table 4:** The prediction performance of the six classification models for PLoS prediction.

**Classifier algorithms**	**Brier score**	**AUC**	**Precision**	**Recall**	**F1-score**	**Accuracy**
LoR	0.182	0.752	0.75	0.75	0.74	0.75
RF	0.226	0.706	0.67	0.68	0.68	0.68
GB	0.181	0.754	0.75	0.75	0.73	0.75
SVM	0.192	0.726	0.74	0.74	0.72	0.74
AdaBoost	0.255	0.643	0.65	0.65	0.65	0.65
KNN	0.198	0.723	0.70	0.71	0.70	0.71

Of the six models, the Gradient Boosting (GB) classifier demonstrated the best prediction performance in terms of accuracy (75.4%), Area Under the Curve (AUC; 0.754), and Brier score (0.181). The Logistic Regression (LoR) model had the second-best performance, with an accuracy of 75%, AUC of 0.752, and a Brier score of 0.182. Based on these results, GB and LoR were chosen as the final models due to their better performance. However, the Ada Boost model showed poor performance with the highest Brier score, lowest accuracy, and lowest AUC values. Despite attempting hyperparameter optimization, the model's accuracy did not significantly improve.

**Figure 9:**
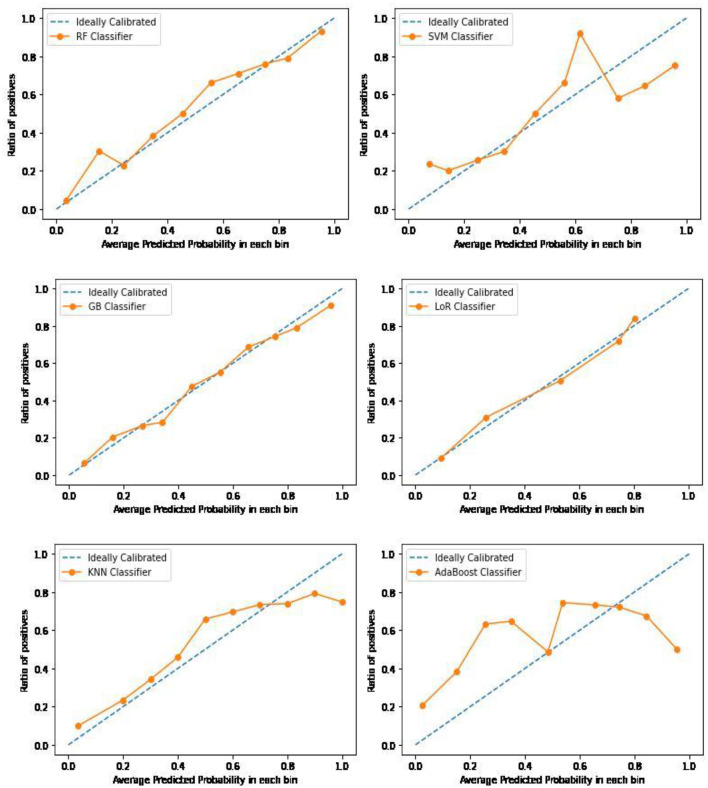
Calibration curve plots of the six classification models for PLoS prediction.

The calibration plots for each model are displayed in Figure 9. The graph shows that GB and LoR have an almost ideal calibration or optimal fit. The Random Forest (RF) and K-Nearest Neighbor (KNN) models are well-calibrated but tend to overestimate the probabilities of a prolonged length of stay (PLoS) for most patients. Conversely, the Ada Boost and Support Vector Machine (SVM) models are poorly calibrated, with Ada Boost underestimating the probability of a PLoS for patients identified as low risk and overestimating it for patients in the two highest risk deciles.

**Figure 10:**
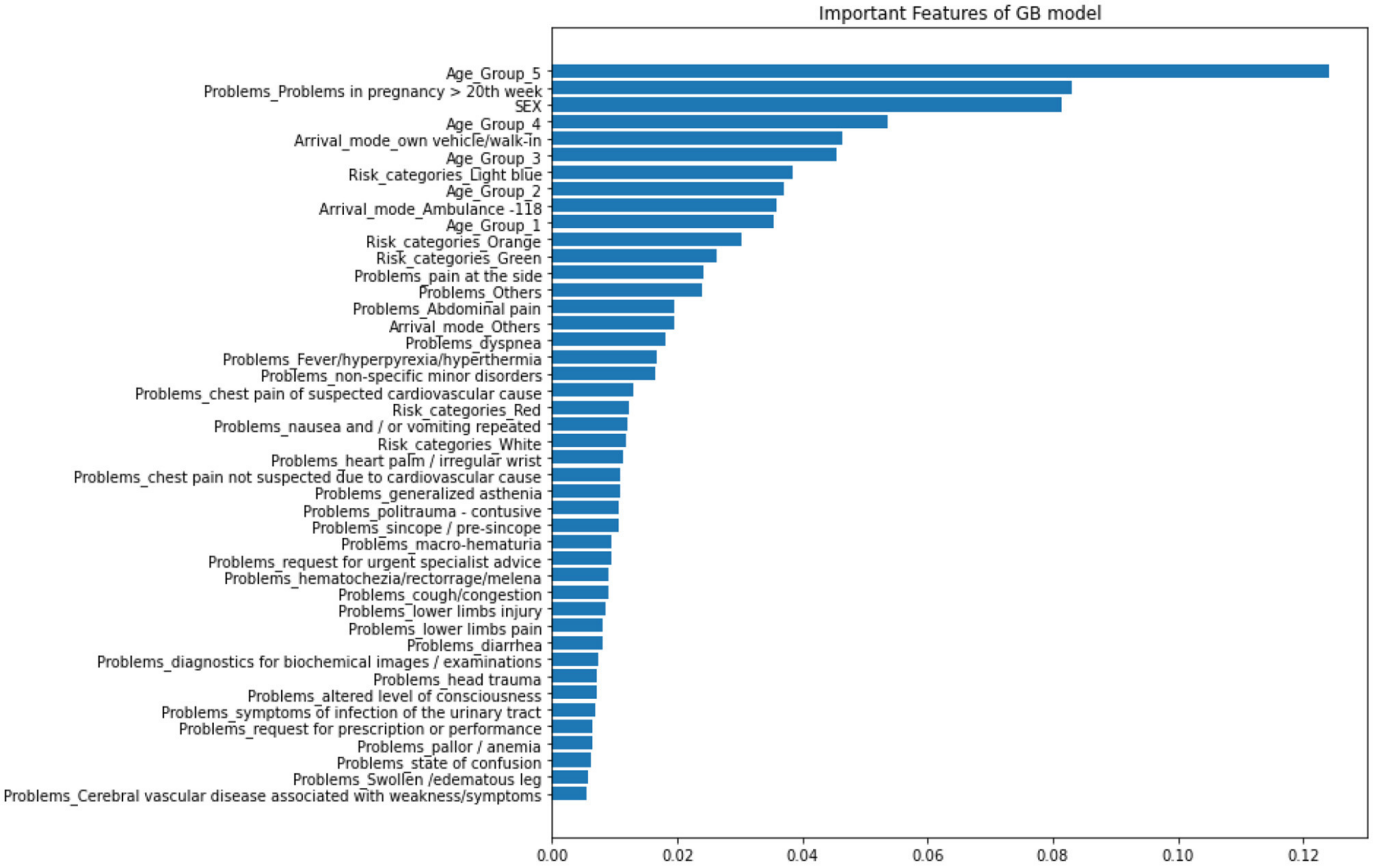
Gradient Boosting variable importance.

The model with the highest prediction accuracy, Gradient Boosting (GB), was used to determine the relative importance of features. Figure 10 displays the results of the variable importance ranking generated by the GB model. In order of importance, the most important features were: Age Group 5 (Individuals over 70 years old), Problems in pregnancy after 20 weeks, Sex, and Age Group 4 (Individuals between 50 and 69 years old).

### 3.4. Prediction and model performance results: continuous outcome

**Figure 11:**
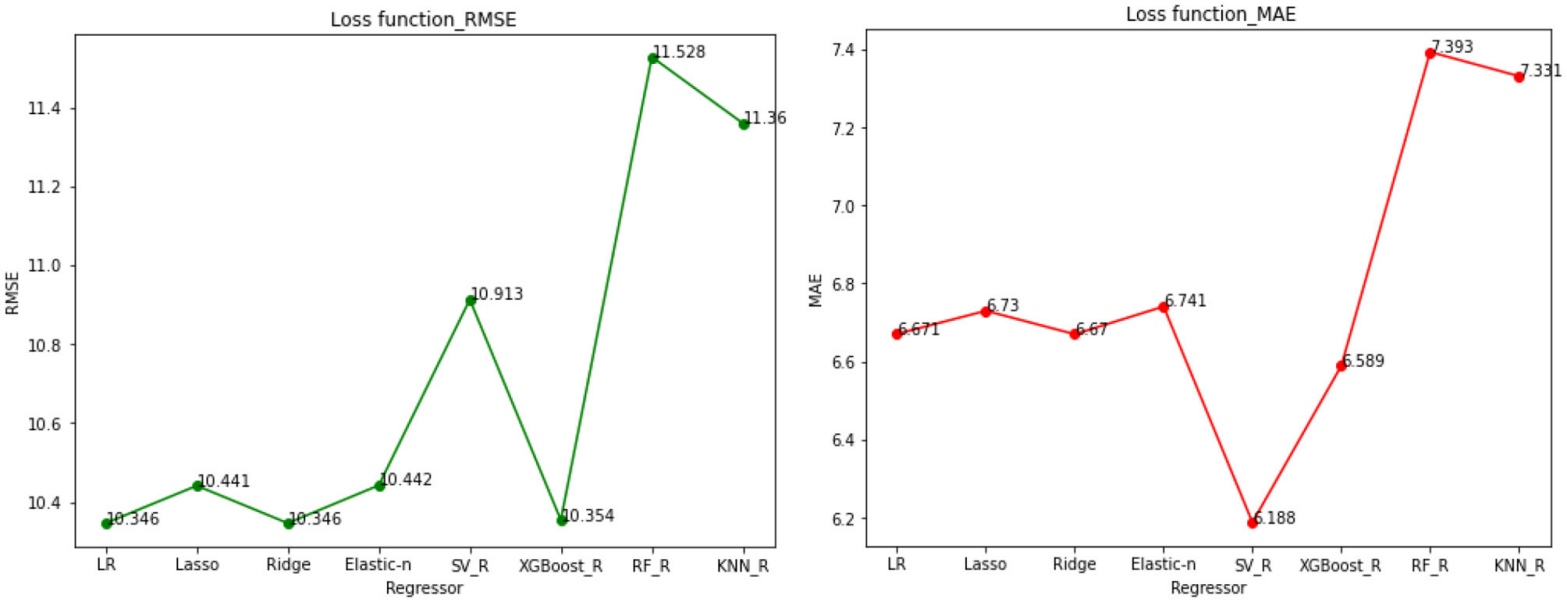
The loss function/total error visualization.

The models used for predicting Length of Stay (LoS) were compared in Table 5, including various linear, penalized linear, and other machine learning models using different loss functions or total error measures. Ridge Regression and XGBoost Regression are identified as the best models based on their lower loss function values. The loss function or the total error performance measure is also visualized in Figure 11, where RMSE is on the left and MAE is on the right.

**Table 5:** Comparisons of classifier methods with continuous target variables for statistical and ML models applied to our datasets.

**Model**	**Loss function**			
	**MSE**	**RMSE**	**MAE**	**MRE**
Linear regression	107.045	10.346	6.671	1.283
*Penalized linear models*	-	-	-	-
Lasso regression	109.034	10.441	6.730	1.319
Ridge regression	**107.044**	**10.346**	**6.670**	**1.283**
Elastic net regression	109.034	10.442	6.741	1.322
*Other ML learning models*	-	-	-	-
Support vector regression	119.103	10.913	6.188	0.854
XGBoost regression	**107.209**	**10.354**	**6.589**	**1.213**
Random forest regressor	132.899	11.528	7.393	1.332
K-nearest neighbors regression	129.045	11.359	7.331	1.315

MSE, mean square error; RMSE, root mean square error; MAE, mean absolute error; MRE, mean relative error.

The bolded values indicate the lowest values of prediction error (e.g. Ridge and XGBoost regressions) for continuous outcomes, LoS.

## 4. Discussion

In this study, we aimed to compare and evaluate predictive models using supervised machine learning algorithms for predicting prolonged length of stay in patients admitted through the emergency department (ED) in general patients settings. It is intended to promote a specific model and suggest or propose a decision-support tool as part of a predictive framework. It is well-established that reducing the length of inpatient hospital stays is one of the ways to improve the quality of life and sustainability of healthcare systems (Baek et al., 2018). Therefore, our study aims to assist physicians and doctors in making informed decisions that enable personalized interventions and guide their decision-making process to predict hospitalizations and enhance healthcare quality.

In most PLoS prediction models, predicting the outcome relies on either classification or regression. Our study utilized two separate modeling methods to predict the outcome, employing both a dichotomous value (PLoS), and a continuous value (LoS)—that is to minimize information loss in a classification task. Adopting precise and accurate modeling techniques improves the results and interpretations. In recent years, the prediction of patient LoS for various diseases and scenarios has been extensively explored using a variety of statistical and machine learning methods such as Logistic Regression (LoR), Random Forest (RF), Support Vector Machines (SVM), K-Nearest Neighbors (KNN), decision tree-based methods, among others (Barsasella et al., 2022).

Of the six classifiers evaluated in this study (LoR, RF, SVM, GB, AdaBoost, and KNN), five of them, excluding AdaBoost, had AUCs > 0.7, suggesting them as effective tools to predict the outcome (Florkowski, 2008). The predictive performance of the classifier models was evaluated using popular statistical indicators such as accuracy, AUC, and Brier score. GB performed the best among the six classifier models, followed by LoR. AdaBoost showed poor performance as it underestimated the probability of PLoS in patients identified as low risk and overestimated it in two patient deciles classified as high risk. Similar results were observed in other studies (Alsinglawi et al., 2020b), which used ML models to predict LoS for adult ICU cardiovascular hospitalizations, with the best results obtained using the GB algorithm.

Several studies, including (Kong et al., 2020; Jo et al., 2021; Wu et al., 2021; Xiong et al., 2022), have shown that the GB classifier outperforms other algorithms in predicting PLoS, with reported accuracy, AUC, and Brier score ranging from 75.3 to 82.9%, 0.74 to 0.873, and 0.122 to 0.156, respectively. Our study's findings are consistent with these results. In contrast to some other studies, Random Forest (RF), a widely used ensemble model, has been shown to perform well in certain contexts. For instance, in Xue et al. (2022), RF achieved high accuracy, AUC, and Brier scores of 0.822, 85.8%, and 0.137, respectively, suggesting its efficacy for predicting length of stay in hospital patients. These findings highlight the importance of carefully selecting the appropriate machine learning algorithm based on the specific data and problem being addressed. While RF may be a strong choice for certain applications, it may not necessarily be the best option in all cases. Therefore, it is crucial to systematically compare the performance of different algorithms and identify the optimal model for a given dataset. Such efforts can ultimately lead to more accurate and reliable predictions for clinical decision-making. Moreover, RF has demonstrated superior performance in predicting the outcome in various healthcare contexts. For example, RF has been shown to perform well in predicting LoS in newborns (Thompson et al., 2018), patients undergoing general surgery (Chuang et al., 2015), and individuals with COPD (chronic obstructive pulmonary disease; Luo et al., 2017). However, the results may vary depending on the specific patient population, clinical variables included in the model, and machine learning algorithm used. Moreover, we analyzed the importance of the features used in our best models, i.e., GB. In order of importance, the most important features were: Age Group 5 (Individuals over 70 years old), Problems in pregnancy after 20 weeks, Sex, and Age Group 4 (Individuals between 50 and 69 years old).

In addition, our study also aimed to predict continuous outcomes using eight ML regression models, as described in the methodology. After evaluating the models' performance, we found that Ridge and XGBoost regressions outperformed the others, resulting in lower prediction errors. Our findings align with previous studies, such as Chen and Klasky (2022), which reported similar results with lower prediction errors or loss functions. For instance, they reported the lowest mean absolute error between prediction and actual duration to be around 4 days, while our study showed a similar result of around 6 days. In addition, the XGBoost regression model also showed better results in Gabriel et al. (2023) for spine surgery LoS prediction. In another study on regression outcomes (Caetano et al., 2014), which examined the general patient population, six regression techniques were compared, including average prediction, decision trees, multiple regression, ANN ensembles, RF, and SVM. The RF regression model was found to yield the most accurate results with the lowest loss. Overall, our study adds to the existing body of literature highlighting the effectiveness of machine learning regression models in predicting continuous outcomes in healthcare. In particular, our results demonstrate the potential of Ridge and XGBoost regressions in improving the accuracy of LoS prediction.

To summarize, selecting the most appropriate ML algorithm that matches the specific data and problem at hand and comparing the performance of different algorithms are crucial steps in identifying the optimal model for a given dataset to ensure accurate and reliable clinical decisions. The best-performing models can then be selected as the final models. As a result, GB followed by LoR is our best-performing classification model, while Ridge Regression and XGBoost Regression were the regression model choices. These final models can now be utilized to make informed decisions or derive meaningful insights for future patients. It is important to note that the choice of the optimal model may depend on various factors, such as the type of data, the problem being addressed, and the specific goals of the analysis. Therefore, it is recommended to evaluate and compare the performance of different models when developing predictive models for various clinical applications.

One of the strengths of our study was that we used all data from ED-admitted patients, so heterogeneous patients were included in the analysis. Moreover, we evaluated several ML techniques for predicting both a categorical and a continuous outcome. However, our study has some limitations that should be recognized. One limitation of the study is that vital signs for triage evaluation information and laboratory test results were not available, which is probably one of the most important indicators (Calzavacca et al., 2010); and data was only collected from one hospital so we were not able to validate the prediction model externally. Moreover, the results of this study may be biased toward other normative periods since the data were collected during the COVID-19 pandemic. Furthermore, interpreting ML results can be difficult due to the black-box nature of some models, which can make it challenging to understand the factors that contribute to the final prediction. However, linear models such as LASSO, Ridge, Elastic-Net Regression, and Logistic Regression provide regression coefficients, making them transparent and easily interpretable (Kotsiantis et al., 2006; Deo, 2015). Other techniques like feature selection and model-agnostic interpretability methods can also improve transparency.

In future work, we will focus on a specific specialty or disease that is prevalent in the hospital. In addition, efforts will be made to incorporate missing features such as vital signs in triage evaluation and laboratory test results. The aim is to enhance the dataset by adding more information regarding features and patients to produce better results and tackle more advanced prediction tasks such as Length of Stay (LoS) after surgeries and utilization of critical hospital resources.

## 5. Conclusions

As a result of our research, we have found that ML models are effective in predicting outcomes. Our findings showed that the GB classifier performed best, followed by LoR. These models can be utilized as a decision-support tool to inform healthcare decisions and predict new patient hospitalizations. Additionally, for continuous outcomes, Ridge regression and XGBoost regression displayed the best prediction performance with the lowest total prediction error. Healthcare providers can utilize our models to predict the hospitalization of new patients or to drive quality improvement initiatives. It is worth mentioning that this study is the first of its kind conducted in this hospital and can serve as a reference for future similar studies and provide valuable insights for informed decision-making.

## Data availability statement

The data presented in this article are not publicly available due to ethical restrictions. Requests to access the data should be directed to the corresponding author.

## Ethics statement

The studies involving human participants were reviewed and ethically approved by the Bioethics Committee of the University of Bologna, Italy (approval number: 0058022, February 24, 2023). Written informed consent from the participants' legal guardian/next of kin was not required to participate in this study in accordance with the national legislation and the institutional requirements.

## Author contributions

AZ: methodology, formal analysis, data curation, and writing—original draft preparation. PP: methodology, data curation, and writing—review and editing. PT: data curation and writing—review and editing. RM: methodology, supervision, and writing—review and editing. LC: conceptualization, methodology, supervision, and writing—review and editing. All authors have read and agreed to the published version of the manuscript.
