# Supplementary material for: Machine learning-based prediction of hospital prolonged length of stay admission at emergency department: a Gradient Boosting algorithm analysis
Source: Front Artif Intell. 2023 Jul 28;6:1179226. doi: 10.3389/frai.2023.1179226 (PMC10426288; doi:10.3389/frai.2023.1179226)
Supplement: Supplementary file 1 [file Data_Sheet_1.pdf]

Appendix:

Table S1. Description of independent features and outcomes.

Features	Measure categories
Case ID	A unique ID for identification of inpatients
Demographic	
Age	Patient Age in classes:<br>[0 - 17), (18 - 29), (30 - 49), (50- 69), 70+
Gender	The state of being female = 1 or male = 0
Arrival mode/admission source	Ambulance -118
	Own vehicle/walk-in
	Others
Risk categories – triaging in entrance	Red – Emergency
	Orange – indifferible urgency
	Light blue – differable urgency
	Green – minor urgency
	White – not urgency
Specialty	General medicine, geriatry , astanteria , obstetrics and gynecology , pediatrics , general surgery, infectious and tropical diseases, orthopedics and traumatology, urology, coronary unit, pediatric surgery, gastroenterology, cardiology, intensive care, pneumology, nephrology, oncology, vascular surgery
	Others (damages, details nephrology (enabled for transplantation), neonatology, pediatric oncology, semi-intensive therapy , maxillo facial surgery , hematology, thoracic surgery, ophthalmology , heart surgery , neonatal intensive care , pediatric heart surgery , and dermatology)
Most frequent problems<br><br># problems are not only diseases specific also any other problems.	Dyspnea, abdominal pain, fever/hyperpyrexia/hyperthermia, problems in pregnancy > 20th week, non-specific minor disorders ,chest pain of suspected cardiovascular cause, sincope / pre-sincope, generalized asthenia, politrauma – contusive, pain at the side, nausea and / or vomiting repeated, heart palm / irregular wrist altered level of consciousness, state of confusion, hematochezia / rectorrage / melena, lower limbs injury, cough / congestion, lower limbs pain, chest pain not suspected due to cardiovascular cause, pallor / anemia, request for urgent specialist advice, macro-hematuria, diarrhea, request for prescription or performance, swollen /edematous leg, weakness of extremities / symptoms associated with cerebrovascular

	disease, symptoms of infection of the urinary tract, diagnostics for biochemical images / examinations, head trauma
	Other problems (more than 135 cases, a few in numbers)
PLoS – outcome variable	LoS < 6 / LoS > = 6 days as binary outcome ; LoS [0 to 120 days) as continuous outcome

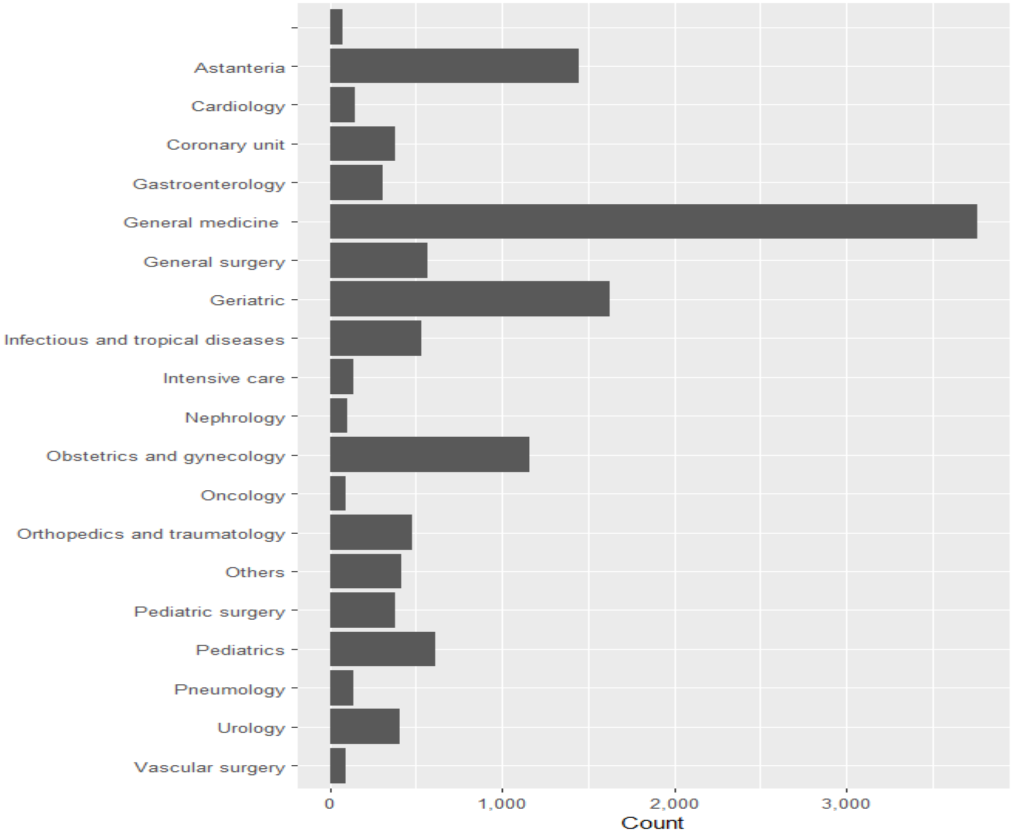

Fig S1. Frequency of type of length of stay (specialty) or Hospital wards during admission patients in 12858 patients the emergency department (ED) of the Sant'Orsola, Malpighi University Hospital of Bologna between January and October 2022.

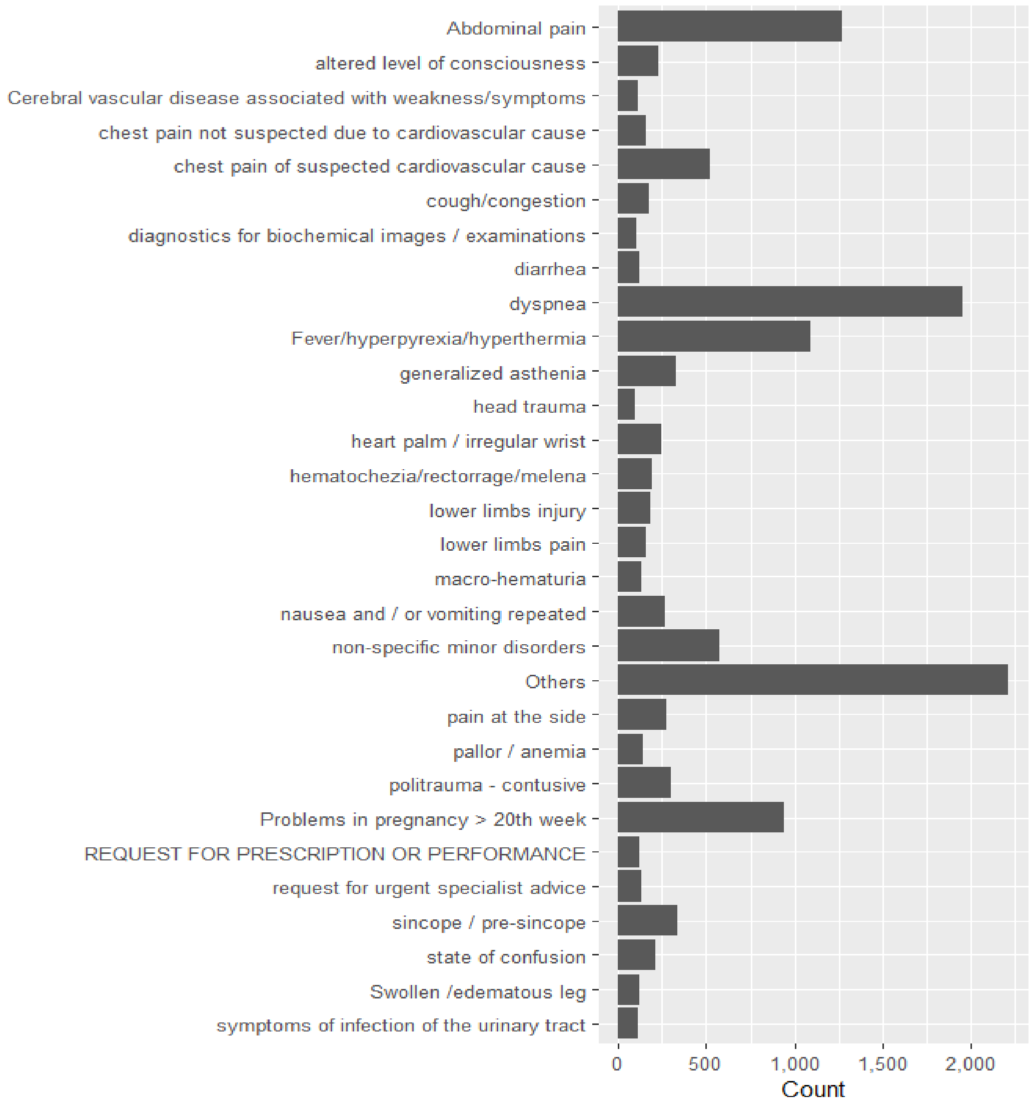

Fig S2. Frequency of most frequent problems during admission or Chief complaint for admission in 12858 patients in the emergency department (ED) of the Sant'Orsola, Malpighi University Hospital of Bologn
